# Exploiting tumor-intrinsic signals to induce mesenchymal stem cell-mediated suicide gene therapy to fight malignant glioma

**DOI:** 10.1186/s13287-019-1194-0

**Published:** 2019-03-12

**Authors:** Man Li, Shoujia Sun, Sean Dangelmajer, Quan Zhang, Junwen Wang, Feng Hu, Fangyong Dong, Ulf D. Kahlert, Mingxin Zhu, Ting Lei

**Affiliations:** 1 Department of Neurology, Tongji Hospital, Tongji Medical College, Huazhong University of Science and Technology, Wuhan, Hubei People’s Republic of China; 2 Department of Neurosurgery and institute of Brain and Brain-Inspired Science, Shandong University, Jinan, People’s Republic of China; 3 Department of Anesthesia, Critical Care, and Pain Medicine, Massachusetts General Hospital, Boston, MA USA; 4 Department of Neurosurgery, Tongji Hospital, Tongji Medical College, Huazhong University of Science and Technology, Wuhan, Hubei 430030 People’s Republic of China; 5 Department of Neurosurgery, University Medical Center Düsseldorf, German Cancer Consortium (DKTK), Essen/Dusseldorf, Germany; 6 Department of Anesthesiology, Tongji Hospital, Tongji Medical College, Huazhong University of Science and Technology, Wuhan, Hubei People’s Republic of China

**Keywords:** Biologic therapy delivery system, Suicide gene therapy, Mesenchymal stem cells, Glioblastoma

## Abstract

**Background:**

Human mesenchymal stem cell (MSC)-based tumor necrosis factor-related apoptosis-inducing ligand (TRAIL) gene delivery is regarded as an effective treatment for glioblastoma (GBM). However, adverse-free target site homing of the delivery vehicles to the tumor microsatellite nests is challenging, leading to erroneously sustained released of this suicide protein into the normal brain parenchyma; therefore, limiting off-target cytotoxicity and controlled expression of the suicide inductor is a prerequisite for the safe use of therapeutic stem cells.

**Methods:**

Utilizing the intrinsic expression profile of GBM and its elevated expression of TGF-β relative to normal brain tissue, we sought to engineer human adipose-derived MSCs (hAMSC-SBE4-TRAIL) which augment the expression of TRAIL under the trigger of TGF-β signaling. We validated our therapeutic technology in a series of functional in vitro and in vivo assays using primary patient-derived GBM models.

**Results:**

Our current findings show that these biologic delivery vehicles have high tumor tropism efficacy and expression TRAIL gene under the trigger of TGF-β-secreting GBMs, as well as avoid unspecific TRAIL secretion into normal brain tissue. hAMSC-SBE4-TRAIL inhibited the proliferation and induced apoptosis in experimental GBMs both in vitro and in vivo. In addition, our improved platform of engineered MSCs significantly decreased the tumor volume and prolonged survival time in a murine model of GBM.

**Conclusions:**

Our results on the controlled release of suicide inductor TRAIL by exploiting an endogenous tumor signaling pathway demonstrate a significant improvement for the clinical utility of stem cell-mediated gene delivery to treat brain cancers. Harvesting immune-compatible MSCs from patients’ fat by minimally invasive procedures further highlights the clinical potential of this approach in the vision of applicability in a personalized manner. The hAMSC-SBE4-TRAIL exhibit great curative efficacy and are a promising cell-based treatment option for GBM to be validated in clinical exploration.

**Electronic supplementary material:**

The online version of this article (10.1186/s13287-019-1194-0) contains supplementary material, which is available to authorized users.

## Introduction

Glioblastoma (GBM) is one of the most common and intractable primary malignant brain tumors, with an annual incidence of 5.26 per 100,000 population or 17,000 new diagnoses per year [1, 2]. Unfortunately, the prognosis of patients with GBM is extremely poor even though current treatments have been expanding and improving. Since GBMs are characterized by leaving behind microscopic tumor satellites into the normal brain parenchyma, tumor recurrences are common despite maximal surgical resection in combination with radiation and chemotherapy [3–5]. Therefore, it is imperative to develop effective therapeutic strategies that focus specifically on targeting and eliminating the disseminated neoplastic burden which have evaded from the main tumor mass.

Recent studies suggested that human stem cells can be used for the delivery of therapeutic genes to treat brain tumors [6–8]. Unlike neural stem cells (NSCs) and induced pluripotent stem cells, human mesenchymal stem cells (MSCs) are easily accessible and are an ethically non-controversial cell resource with high promise in tissue repair and tumor therapy [9–12]. Immunologically identical genetic profiles of MSCS in autologous applications further highlight their therapeutic potential. Several studies reported that human adipose-derived mesenchymal stem cells (hAMSCs) exhibit remarkable tropism specificity toward glioblastoma including the tracking of *hard-to-resect* disseminated microscopic tumor foci [13–15]. Additionally, hAMSCs can effectively be expanded in relatively low-cost culture conditions providing a large scale genetically and morphologically stable cell resource [12, 16]. Together with the possibility to genetically equip MSCs with therapeutic loads, hAMSCs are promising candidates for biological deliveries of therapies to fight a brain tumor.

Tumor necrosis factor-related apoptosis-inducing ligand (TRAIL) is a highly potent therapeutic agent that has been shown to significantly increase the apoptosis as well as decrease the migration capacity of GBM cells [17, 18]. There have been reports that MSC-based TRAIL gene delivery has therapeutic benefit in mouse models GBMs [19–21]. However, a problem with systemic therapies of stem cells secreting TRAIL is that not all of these delivery vehicles reach the tumor micrometastatic nests. Moreover, many of the stem cells home into the normal brain parenchyma and along the perivascular spaces. As a result, the stem cells loaded with TRAIL that do not make it to the tumor satellites erroneously sustain their release into normal brain tissue leading to side effects such as neuronal cell death. Those reasons limit the use of TRIAL secreting therapeutic stem cells in clinical oncology [22–24]. The generation of safe and trackable stem cell delivery systems allowing the selective TRAIL therapy secretion is of highest clinical need.

Recent evidence indicates that, compared with normal brain tissue, up to 70% of gliomas, especially GBM, secrete high levels of TGF-β to maintain self-renewal, invasion, and tumorigenicity of glioma stem cells [25–28]. Here, we sought to develop biologically engineered hAMSC-SBE4-TRAIL to express TRAIL through TGF-β signaling via a SMAD4-controlled minimal promoter. Since GBM express TGF-β more robustly than non-cancer cortex, we hypothesize one can exploit this discrepancy as an inducer to activate the therapeutic molecules release from hAMSC-SBE4-TRAIL. Our results demonstrate that, compared to MSCs with sustained secretion of TRAIL, hAMSC-SBE4-TRAIL exhibit lower off-target complication and more comprehensive cancer cell targeting capacity. These results are paramount to understanding the utility of hAMSC-SBE4-TRAIL to treat GBM patients in whom tumor mass express an elevated level of TGF-β. Given the individual genetic and epigenetic profile of signaling network activation of each patient’s tumor, as well as the broad collection well-studied suicide inducing strategies, we hypothesize that this model can be adapted on individual cases to provide a highly tailored, custom-made biologic treatment in oncology and contribute to the utility of stem cells in personalized medicine.

## Materials and methods

### Cell lines

Following the approval by the Huazhong University of Science and Technology (HUST) Institutional Review Board, early passaged primary human adipose-derived mesenchymal stem cells (hAMSCs 173) and human glioblastoma (GBM025, GBM079, GBM106) were obtained from patients undergoing neurosurgical procedures as described in our previous studies [7, 15, 29]. The hAMSCs were cultured in MSC complete media [MesenPRO RS basal media with one vial of MesenPRO RS growth supplement (GIBCO), 1% penicillin/streptomycin (GIBCO), and 1% GlutaMAX (GIBCO)]. The GBM cells were cultured in GBM complete media [DMEM/F12 (GIBCO) with 10% fetal bovine serum (Invitrogen), 1% penicillin/streptomycin (GIBCO), and 1% GlutaMAX (GIBCO)]. Commercial human astrocyte (ScienCell, #1800) was cultured in astrocyte complete media [DMEM/F12 (GIBCO) with 10% fetal bovine serum (Invitrogen), 1% penicillin/streptomycin (GIBCO), and 1% GlutaMAX (GIBCO)]. Cells were incubated at 37 °C in a humidified atmosphere containing 5% CO_2_.

### Lentiviral production, infection, and identification

**Fig. 1:**
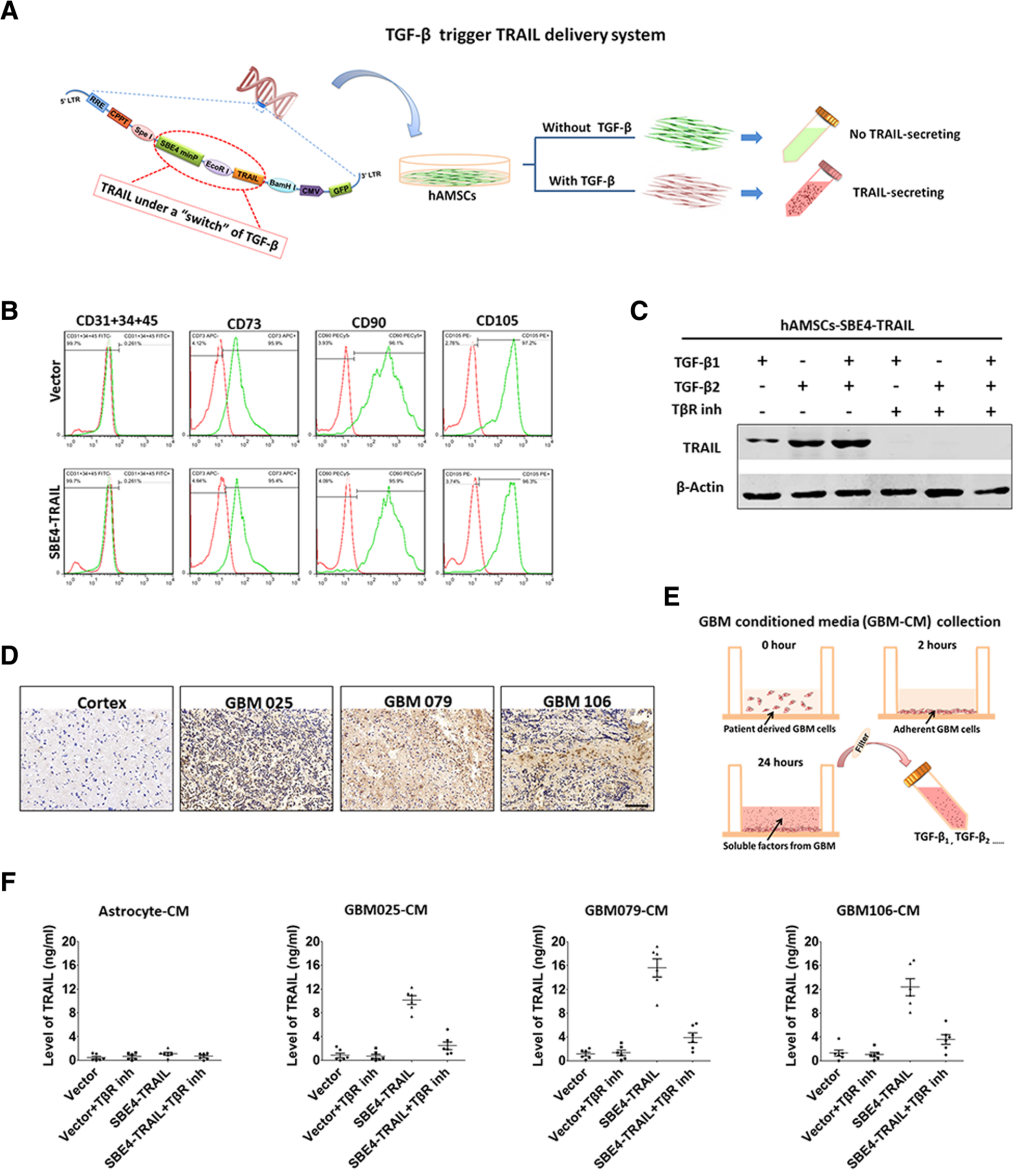
Primary hAMSCs engineered to express TRAIL under the control of TGF-β. **a** Schematic of SBE4TRAIL-duet lentiviral and hAMSC-SEB4-TRAIL production (TGF-β trigger TRAIL delivery system). **b** Flow cytometric analysis was performed to confirm that primary hAMSC-SBE4-TRAIL expressed high level of CD73, CD90, and CD105. Meanwhile, CD31, CD34, and CD54 positive cells were absent in these stem cells. **c** Western blots were performed to test the TRAIL expression of hAMSC-SBE4-TRAIL presence or absence of extrinsic TGF-β1 and/or TGF-β2 at 10 ng/ml with or without TGF-β receptor inhibitor (LY2109761, Eli Lilly). **d** Immunohistochemistry staining was used to determine the TGF-β level in the normal cortex and glioblastoma tissues. Scale bar, 100 μm. **e** Schema showing the collection of these primary patient-derived GBM cells conditioned media (GBM-CM). **f** The concentration of TRAIL in hAMSC-vector and hAMSC-SBE4-TRAIL that were pretreated in the astrocyte-CM and three GBM-CM for 24 h was measured using an ELISA kit

To identify hAMSCs, astrocyte, and GBM in our in vitro and in vivo experiments, we transduced these cells with lentiviral vectors coding for GFP, GFP/Fluc, td-tomato, or td-tomato/Fluc (Vigene Biosciences). Lentiviral vector-driven expression of TRAIL in response to TGF-β (SBE4TRAIL-Duet) was used to transduce the hAMSCs (Fig. 1a). To confirm TGF-β controlled hAMSCs expressing TRAIL, hAMSC-SBE4-TRAIL or hAMSC-vector were cultured in MSC complete media in the presence or absence of TGF-β1 (Abcam) and/or TGF-β2 (Abcam) at a concentration of 10 ng/ml. TRAIL expression was assessed by Western blot and ELISA (BOSTER). Viral vector was packaged from HEK293 cells. After collection, astrocyte (astrocyte-td-tomato), GBMs (GBM-td-tomato, GBM-td-tomato/Fluc), and hAMSCs (hAMSC-vector-GFP, hAMSC-vector-GFP/Fluc, hAMSC-SBE4-TRAIL-GFP, hAMSC-SBE4-TRAIL-GFP/Fluc) were sorted by a MoFlo cytometer (Beckman Coulter).

### In vitro studies

MTT, EdU, and Ki67 staining were performed to evaluate GBM cell proliferation in response to hAMSC-SBE4-TRAIL. GBM025, GBM079, and GBM106 were co-cultured with commercial hAMSCs or these delivery vehicles (hAMSC-vector, hAMSC-SBE4-TRAIL), or hAMSC-SBE4-TRAIL under the exposure of TGF-β receptor inhibitor LY2109761 (Eli Lilly) for 24 h respectively. Then, the co-culture conditioned media of each group (control-C-CM, vector-C-CM, SBE4-TRAIL-C-CM, and SBE4-TRAIL+TβR inh-C-CM) were collected (Fig. 3a). Next, the cells were incubated with EdU reagent for 4 h (Invitrogen) and quantified via flow cytometry. 2 × 10^4^ GBM cells, which were cultured in different co-culture conditioned media, were seeded on a 24-well plate for 48 h. The number of Ki67-positive cells from nine random fields was visualized and recorded with an inverted fluorescence microscope (Zeiss).

FACS-based annexin V and propidium iodide (PI) positivity quantification were used to identify the onset of apoptosis (ThermoFisher). The analysis was performed 12 h after treatment. Three replicate experiments were performed for each group. Moreover, GBM025, GBM079, and GBM106 were co-cultured with hAMSC-vector (vector), hAMSC-SBE4-TRAIL (SBE4-TRAIL), and hAMSC-SBE4-TRAIL plus TGF-β receptor inhibitor for 24 and 48 h (SBE4-TRAIL+TβR inh). The percentages of GFP and td-tomato labeled cells as well as the level of cleaved caspase-8 and cleaved caspase-3 to quantify survival/apoptosis of GBM cells in each group were assessed.

The concentration of TGF-β1, TGF-β2, and TRAIL in different conditional media were measured using Abcam’s TGF-β1 (ab100647), TGF-β2 (ab100648) ELISA kit, and Boster’s TRAIL ELISA kit (EK0532), respectively. All measurements were performed according to the recommended protocol. Then, the cells pretreated with different conditions were lysed with NP40 buffer supplemented with protease (Sigma) and phosphatase inhibitors (ThermoFisher). The harvested proteins from each group were resolved on 10% SDS-PAGE, immunoblotted with antibodies against TRAIL (1:1000, Abcam), caspase-3 (1:1000, Abcam), cleaved caspase-3 (1:500, Cell Signaling Technology), caspase 8 (1:1000, Abcam), cleaved caspase 8(1:1000, Cell Signaling Technology), or β-actin (1:2000, Abcam) and detected by chemiluminescence after incubation with horseradish peroxidase-conjugated secondary antibodies.

### In vivo studies

Male athymic nude mice were used in accordance with the ethical guidelines set by Huazhong University of Science and Technology (HUST) and relevant sections of the ARRIVE Guidelines were followed. To determine the capacity of hAMSCs to migrate toward GBM cells, 2 × 10^5^ GBM079-td-tomato (*n* = 6) or 2 × 10^5^ astrocyte-td-tomato (*n* = 6) were injected into the right striatum (AP = − 0 .6 mm, *R* = 3 .5 mm, *V* = 3 .7 mm). One week post injection, 2 × 10^5^ hAMSC-SBE4-TRAIL-GFP were injected into the right striatum (AP = − 0 .6 mm, *R* = 2 .5 mm, *V* = 3 .2 mm). After 1 week, the mice brains were perfused, sectioned at a 20-um thickness, then the migration number and distance of hAMSCs (GFP+/DAPI+) was calculated using ImageJ.

To identify the safety of hAMSC-SBE4-TRAIL into the normal brain, 2 × 10^5^ hAMSC-SBE4-TRAIL-GFP/Fluc were suspended in 5 ul MSC complete media and stereotactically injected into the right striatum of nude mice (AP = − 0 .6 mm, *R* = 2 .7 mm, *V* = 3 .2 mm, *n* = 3). Following injection, these animals were imaged using an IVIS small animal imaging system (Perkin Elmer) at different time periods (2, 4, 6, and 8 weeks post injection). Then, the mice brains were perfused, fully cryo-sectioned at a 20-um thickness. TRAIL+ (Abcam)/GFP/DAPI, cleaved caspase 8+ (Cell Signaling Technology)/GFP/DAPI, and cleaved caspase-3+ (Cell Signaling Technology)/GFP/DAPI were used to stain and measure the effect in the normal brain. The mice with or without hAMSC-SBE4-TRAIL implanted were followed for 10 weeks to record body weight and for 90 days to monitor survival (control, *n* = 8; SBE4-TRAIL, *n* = 8). Kaplan-Meier survival analysis was performed with results reported as the median and mean survival times with a 95% confidence interval.

To assess the anti-GBM therapy efficacy of hAMSC-SBE4-TRAIL, 5 × 10^5^ GBM079-td-tomato were injected into the right striatum in each group (AP = − 0 .6 mm, *R* = 3.0 mm, *V* = 3 .5 mm). After a week, 5 × 10^5^ hAMSC-vector-GFP (vector, *n* = 6), 5 × 10^5^ hAMSC-SBE4-TRAIL-GFP (SBE4-TRAIL, *n* = 6), 5 × 10^5^ hAMSC-SBE4-TRAIL-GFP + TβR inhibitor (SBE4-TRAIL+TβR inh, *n* = 6), or equal volume of PBS (control, *n* = 6) were injected into the same location of tumor mass in right striatum (AP = − 0 .6 mm, *R* = 3 .0 mm, *V* = 3 .5 mm). Two weeks post injection, the mice brains were perfused, sectioned at a 20-um thickness. Then, the migration number and distance of GBM cells, as well as the volume of tumor mass were assayed. Moreover, the number of TRAIL, cleaved caspase 8, and cleaved caspase-3 positive cells of tumor satellites (beyond the border of tumor mass) in SBE4-TRAIL and SBE4-TRAIL+TβR inhibitor group was calculated based on 20 slides per group. The average distance from the center of hAMSC/GBM mass to each GFP/td-tomato positive cell was calculated. A minimum of 150–200 migrating cells were counted for each group. These data were analyzed with ImageJ.

The engineered hAMSC is regarded as a potential strategy which could be exploited for human application in a clinical trial, especially for the patient whom suffered by brain cancer recurrence. In this study, we investigated the different therapeutic approaches to simulate the clinical delivery methods. Intracardiac injection simulated the interventional chemotherapy with artery system for brain tumors. And intracranial injection was just like a stereotactic surgery. Meanwhile, intrathecal injection simulated the intrathecal chemotherapy with cerebrospinal fluid system. Firstly, 5 × 10^5^ GBM079-td-tomato/Fluc were injected into the left striatum of the models (AP = − 0 .6 mm, *L* = 3.0 mm, *V* = 3 .5 mm; control, *n* = 20; intracranial, *n* = 20; intracardiac, *n* = 20; intrathecal, *n* = 20). Seven days post tumor cells injection, 5 × 10^5^ hAMSC-SBE4-TRAIL were injected by intracranial, intracardiac, intrathecal, or sham injection in the various groups. These animals (4/20 in each group) were imaged using an IVIS small animal imaging system (Perkin Elmer) at different time periods (1, 10, 20, and 30 days post tumor cells injection). Fifteen days post hAMSC injection, the mice brains were perfused (6/20 in each group), sectioned at a 20-um thickness, then the average volume of tumor mass was calculated using ImageJ. To further validate the effects mediated against the tumor by each delivery method, the other mice (10/20 in each group) were followed for 90 days to monitor survival. Kaplan-Meier survival analysis was performed with results reported as the median and mean survival times with a 95% confidence interval.

### Statistical analysis

All data are expressed as mean ± SEM. Data within the two groups were analyzed with the Student’s *t* test. One-way analysis of variance followed by the Tukey post hoc test was used to evaluate multiple groups. Statistical significance was defined as *P* < 0.05.

## Result

### Engineered primary hAMSCs express TRAIL under the control of TGF-β-secreting primary GBMs

hAMSCs were efficiently transduced with an engineered lentiviral vector expressing TRAIL in response to TGF-β (Fig. 1a). In order to examine stem cell characteristics of the cells, cell surface marker expression and Vimentin staining were performed. Both hAMSC-vector and hAMSC-SBE4-TRAIL remained negative for CD31, CD34, and CD45 and expressed high levels of CD73, CD90, CD105, and Vimentin (Fig. 1b and Additional file 1: Figure S1A–C). To confirm TGF-β controlled expression of TRAIL, hAMSC-SBE4-TRAIL were cultured in MSC complete media in the presence or absence of TGF-β1 and TGF-β2 for 10 ng/ml. As shown in Fig. 1c, the hAMSC-SBE4-TRAIL induce the expression of TRAIL under the exogenous treatment with TGF-β. Next, TGF-β-secreting primary GBM cells were used to see if they could cause similar TRAIL-induced expression in MSCs. Firstly, the immunohistochemistry staining demonstrated that these GBM tissues (GBM025, GBM079, and GBM106) have higher levels of TGF-β compared with normal cortex (Fig. 1d). Then, primary patient-derived GBM cells were cultured (Additional file 2: Figure S2A), and the GBM conditioned media (Fig. 1e) and astrocyte conditioned media were collected. As shown in the ELISA assessment (Additional file 2: Figure S2B and C, *p* < 0.05), the conditioned media from GBM025, GBM079, and GBM106 cells also displayed higher levels of TGF-β_1_ and TGF-β_2_ than those of normal astrocytes. Next, the hAMSC-vector and hAMSC-SBE4-TRAIL were pre-cultured in astrocyte conditioned media (astrocyte-CM), and GBM conditioned media (GBM025-CM, GBM079-CM and GBM106-CM) for 24 h with or without TGF-β receptor inhibitor (TβR inh). Western blots (Additional file 3: Figure S3A–D) and ELISA (Fig. 1f) results indicated that hAMSC-SBE4-TRAIL induce the expression of TRAIL under the control of TGF-β-secreting GBMs. This effect can be specifically neutralized by introducing TβR inh in the media of the GBM cells. Taken together, the results demonstrate that the engineered hAMSCs induce TRAIL secretion in response to stimulation by exogenously derived TGF-β signaling, thereby precisely controlling the release of the suicide gene payload.

### hAMSC-SBE4-TRAIL display safety and a great capacity of migratory to GBM in vivo

**Fig. 2:**
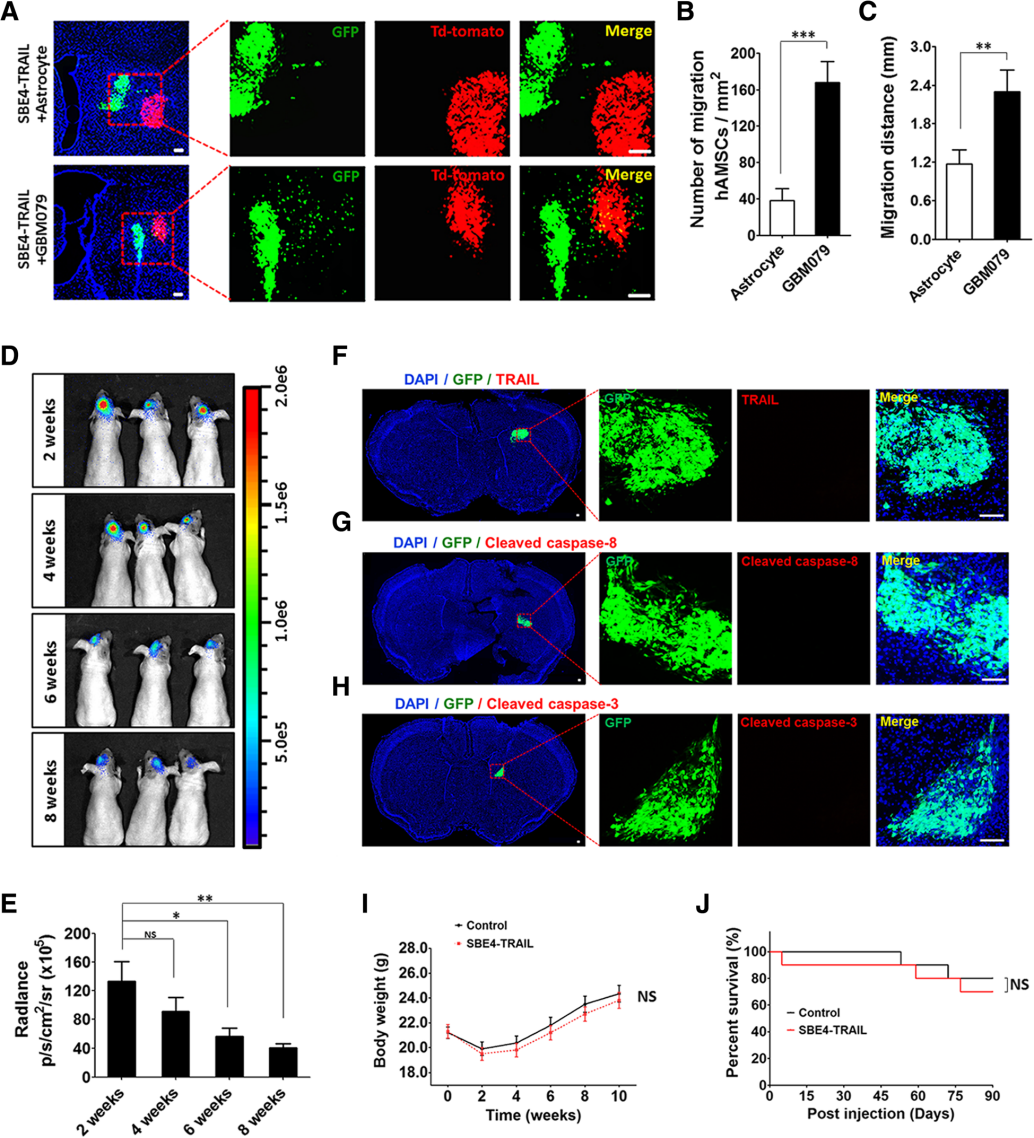
hAMSC-SBE4-TRAIL display tropism toward GBM and limited toxicity in animal models. **a** Mice brain sections were immunostained to test the tropism of hAMSC-SBE4-TRAIL (SBE4-TRAIL, GFP) for GBM (Td-tomato) or astrocyte (Td-tomato) in vivo. **b**, **c** hAMSC-SBE4-TRAIL displayed the higher number and longer distance toward GBM tumor bulk when compared with astrocyte group. Scale bar, 200 μm. **d** Bioluminescence for the hAMSC-SBE4-TRAIL bearing mice was checked on weeks 2, 4, 6, and 8. **e** Bioluminescence radiance was maintained between week 2 and week 4 for hAMSC-SBE4-TRAIL, whereas an obviously decrease in week 6 and week 8. **f**–**h** Representative images showed the apoptotic effects on the normal brain tissue. There were no positive stains for TRAIL, cleaved caspase-8 and cleaved caspase-3 in the normal brain tissue of animal models. Scale bar, 100 μm. **i**, **j** Body weight and survival percentage assay displayed no significant different between hAMSC-SBE4-TRAIL bearing mice and the mice in control group (*n* = 8 per group). Error bars represent SEM. *p < 0.05, **p < 0.01, ***p < 0.001; NS not significant

To determine the tropism of hAMSCs in a mouse model of GBM, 2 × 10^5^ GBM-td-tomato (GBM079, *n* = 6) or 2 × 10^5^ astrocyte-td-tomato (*n* = 6) were injected into the right striatum of immune compromised rodent hosts. One week post injection, 2 × 10^5^ hAMSC-SBE4-TRAIL were implanted at a 1-mm distance from the grafts (Fig. 2a). Immunohistofluorescence staining indicated that there was a greater number as well as greater distance migrated by hAMSC-SBE4-TRAIL toward GBM cells, whereas few hAMSC-SBE4-TRAIL were seen within the astrocyte transplant group (Fig. 2b, c. no. of migratory cells, astrocyte vs. GBM: 49.2 vs. 168.4, *p* < 0.001, migration distance, astrocyte vs. GBM: 1.1 18 mm vs. 2.2 96 mm, *p* < 0.01). To avoid unintended long-term off-target effects by implanted MSCs, we longitudinally assessed their phenotypic properties upon transplantation by in vivo imaging. The hAMSC-SBE4-TRAIL-GFP/Fluc were implanted intracranially as described and animals imaged starting from week 2 to 8 post injection. As shown in Fig. 2d, the bioluminescent signal was declined gradually after 6 weeks which indicated a spontaneous decline of surviving hAMSCs in vivo (Fig. 2e, 2 weeks vs. 6 weeks, *p* < 0.05; 2 weeks vs. 8 weeks, *p* < 0.01). In order to evaluate hAMSC-SBE4-TRAIL inducing apoptosis effects on themselves and normal brain tissue, the amount of excretion of TRAIL, activation of cleaved caspase-8, and cleaved caspase-3 were examined. Strikingly, there was no detection of any hAMSC-derived TRAIL signals in these non-tumorigenic control experiments (Fig. 2f). Moreover, hAMSCs did not display signals consistent with apoptosis nor did was significant cell death observed in normal brain cells. (Fig. 2g, h). Additionally, animals did not display detectable significant clinical effects upon receiving the transplants as no significant loss of body weight occurred (Fig. 2i, *p* > 0.05) nor did any impact on overall percent survival (Fig. 2j, *p* > 0.05) when comparing the hAMSC-SBE4-TRAIL injection group to the control. These studies demonstrate that our engineered primary hAMSC-SBE4-TRAIL have good tropism toward GBM and have minimal risk of toxicity on non-cancer cells in the brain in vivo.

### hAMSC-SBE4-TRAIL decreased proliferation of GBM in vitro

**Fig. 3:**
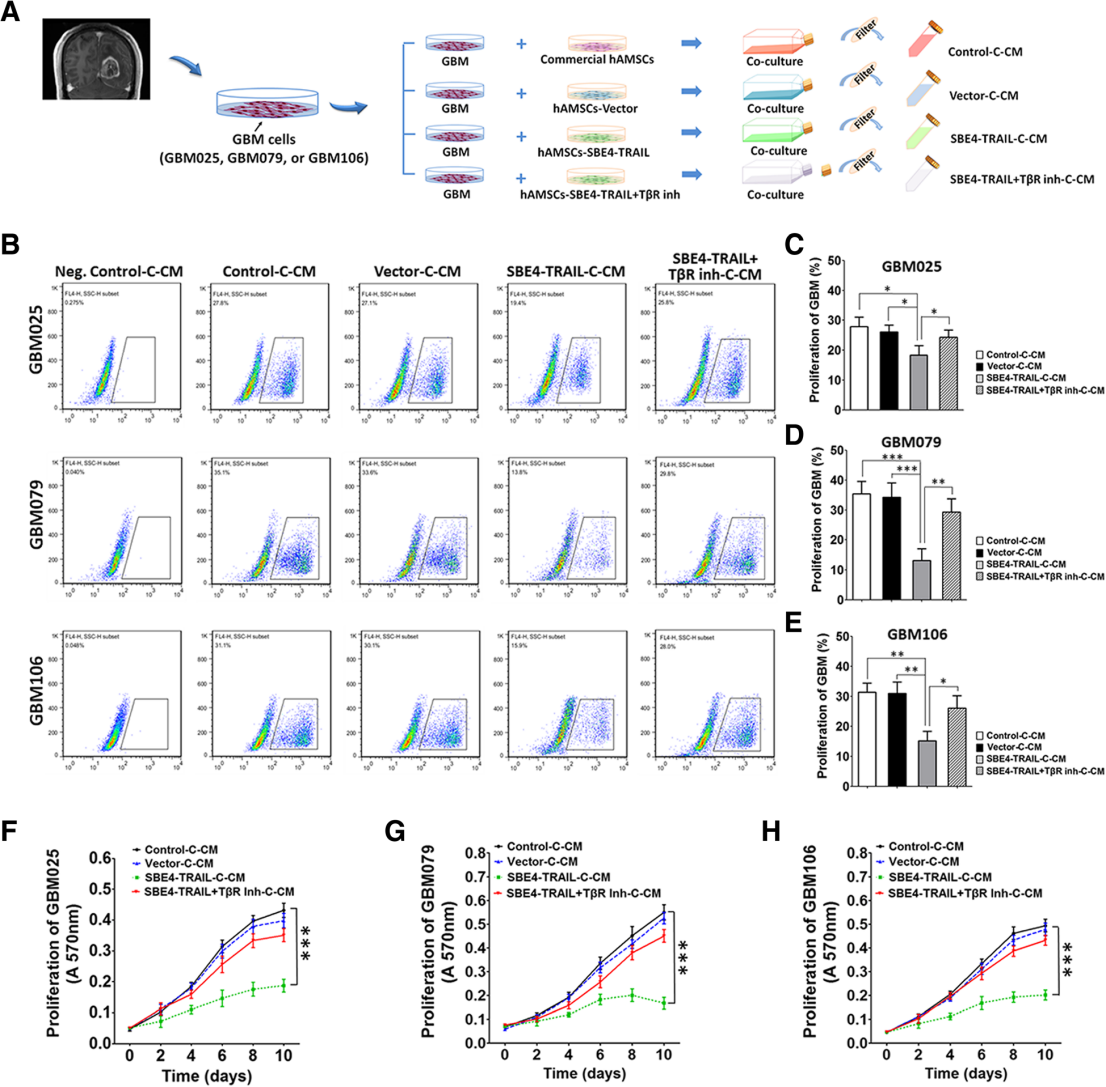
hAMSC-SBE4-TRAIL decreased proliferation of GBM in vitro. **a** Schema showed the collection of co-cultured conditioned media of GBM with commercial hAMSCs, hAMSC-vector, hAMSC-SBE4-TRAIL, or hAMSC-SBE4-TRAIL+TβR inh (control-C-CM, vector-C-CM, SBE4-TRAIL-C-CM, SBE4-TRAIL+TβR inh-C-CM). **b**–**e** Flow cytometry of Edu assay showed that the GBM cells (GBM025, GBM079, and GBM106) cultured in SBE4-TRAIL-C-CM had a significant decrease in proliferation compared to those cultured in other groups. **f**–**h** MTT assay quantification of GBM025, GBM079, and GBM106 exhibited that these patient-derived GBM cells cultured in SBE4-TRAIL-C-CM displayed a decreased rate of proliferation when compared with those cultured in control-C-CM conditions. Error bars represent SEM. *p < 0.05, **p < 0.01, ***p < 0.001

Edu and MTT assays as well as Ki67 staining were used to evaluate the effects of hAMSC-SBE4-TRAIL on GBM cell growth and proliferation. Primary tumor cells (GBM025, GBM079, GBM106) were co-cultured for 24 h with commercial hAMSCs, hAMSC-vector, hAMSC-SBE4-TRAIL, and hAMSC-SBE4-TRAIL+TβR inh, respectively, to collect the co-cultured conditioned media (C-CM) (Fig. 3a). Cell lines GBM025, GBM079, and GBM106 which were cultured in SBE4-TRAIL-C-CM displayed decreased proliferation capacity when compared with control-C-CM group (Fig. 3b–h). Additionally, GBM025, GBM079, and GBM106 also displayed a significant decreasing of Ki67 positive cells in SBE4-TRAIL-C-CM condition while compared with other groups (Additional file 4: Figure S4A–D, fold change in SBE4-TRAIL-C-CM: GBM025, 0.414; GBM079, 0.275; GBM106, 0.332). These results show that hAMSC-SBE4-TRAIL was capable of decreasing the proliferation of primary GBM in vitro.

### hAMSC-SBE4-TRAIL-induced apoptosis in human primary GBM cells in vitro

**Fig. 4:**
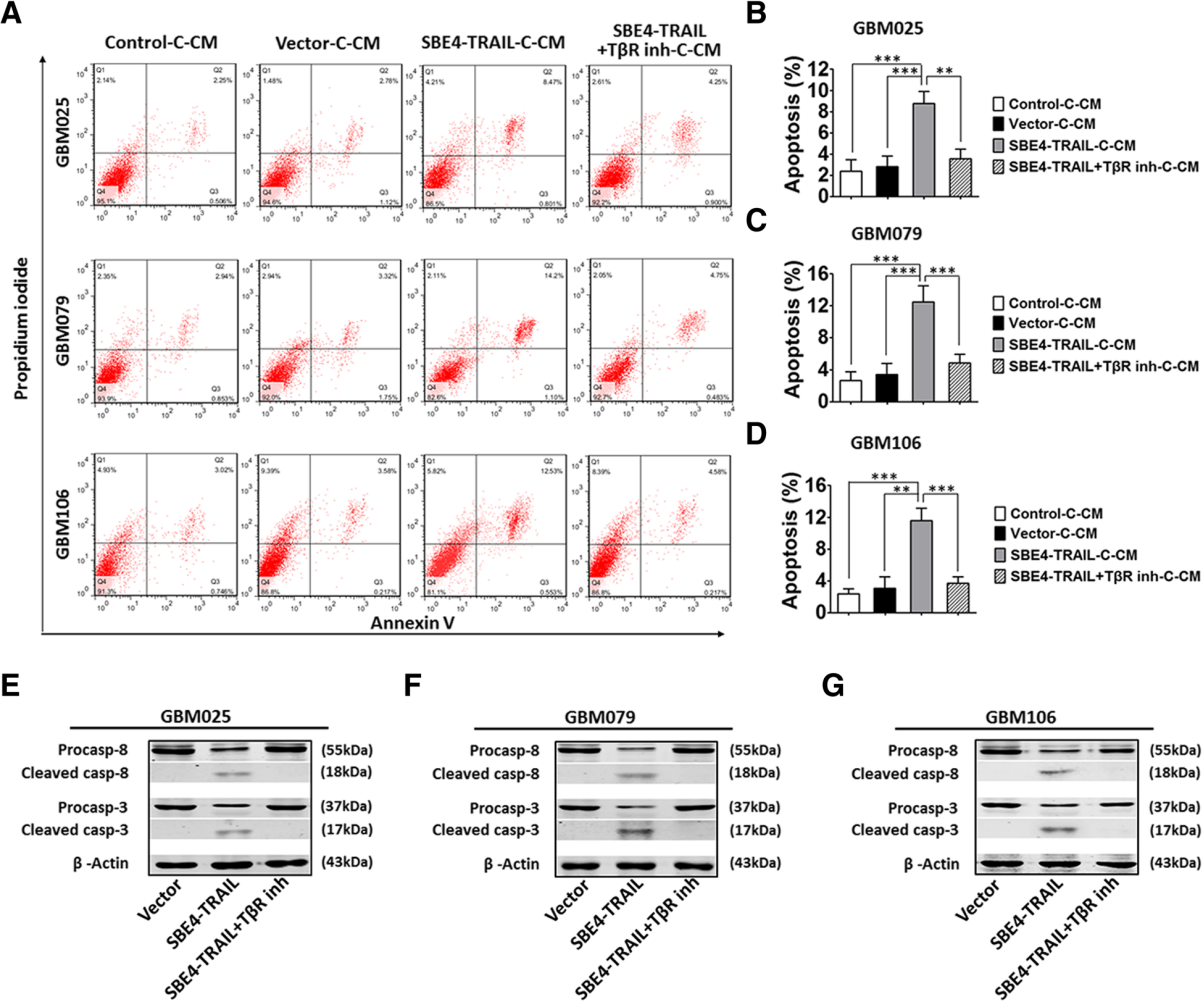
hAMSC-SBE4-TRAIL increased apoptosis in human primary GBM in vitro. **a** Representative images showed the flow cytometry of annexin V and PI assay. **b–d** The GBM cells (GBM025, GBM079, and GBM106) cultured in SBE4-TRAIL-C-CM displayed a significant increasing in apoptosis than cultured in control-C-CM, vector-C-CM, and SBE4-TRAIL+TβR inh-C-CM conditions. **e**–**g** Western blot assay cleaved caspase-8 and cleaved caspase-3 were detected in the SBE4-TRAIL group, but were essentially absent in vector and SBE4-TRAIL+TβR inh group. GBM025, GBM079, and GBM106 co-cultured with SBE4-TRAIL exhibited a higher level of cleaved caspase-8 as well as cleaved caspase-3 when compared with other groups. Error bars represent SEM. *p < 0.05, **p < 0.01, ***p < 0.001

To quantitatively assess the cytotoxic effects of hAMSC-SBE4-TRAIL on GBM cells, annexin V and PI staining were used to assess apoptosis. GBM cells which were pretreated with SBE4-TRAIL-C-CM displayed a significantly higher level of annexin V and PI staining when compared with control-C-CM groups (Fig. 4a–d). To verify these results, GBM025, GBM079, and GBM106 each were co-cultured with hAMSC-vector, hAMSC-SBE4-TRAIL, or hAMSC-SBE4-TRAIL+TβR inh for 0.5, 24, and 48 h, respectively. As shown in the results, when GBM cells were co-cultured with hAMSC-SBE4-TRAIL, the percentage of GBM (Td-tomato/Td-tomato+GFP) was significantly decreased over time as compared to other groups (Additional file 5: Figure S5A–L). Similarly, caspase-3 and caspase-8 activation was increased in a similar manner when co-cultured with TRAIL containing CM (Fig. 4e–g). These experiments demonstrate that the secretome of hAMSC-SBE4-TRAIL induces apoptosis of GBM in vitro.

### hAMSC-SBE4-TRAIL inhibit growth and induce apoptosis in human GBM cells in vivo

**Fig. 5:**
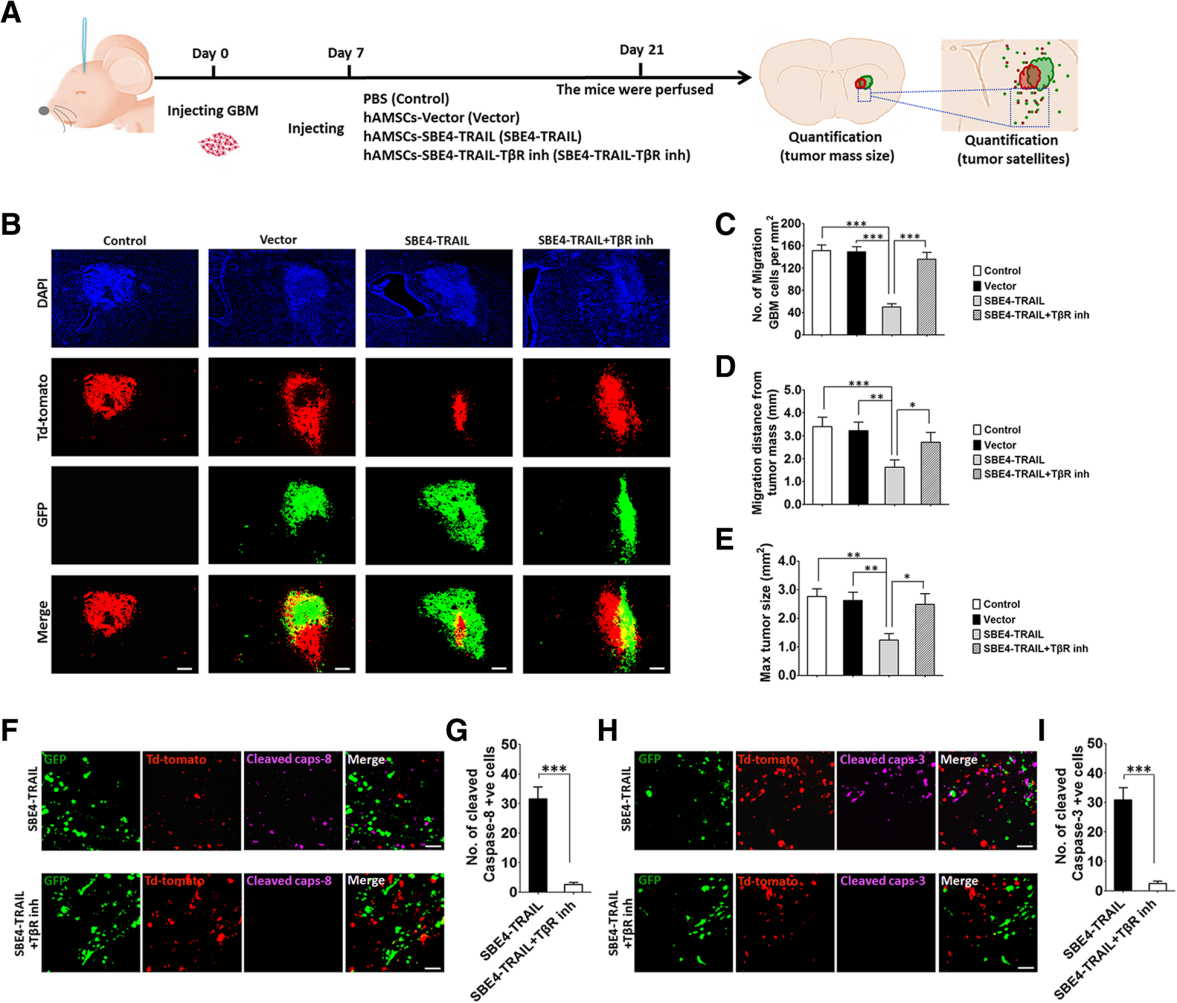
hAMSC-SBE4-TRAIL inhibit growth and induce apoptosis in human GBM in vivo. **a** Schematic of the injection experiment where different delivery vehicles (PBS, hAMSC-vector, hAMSC-SBE4-TRAIL, or hAMSC-SBE4-TRAIL+TβR inh) were injected into the same location of tumor mass of GBM bearing mice, which sacrificed 2 weeks later. **b** Representative images showed the treatment efficacy of hAMSCs (GFP, green) in GBM (Td-tomato, red) bearing mice. Scale bar, 200 μm. **c**, **d** The migration of GBM cells assay showed hAMSC-SBE4-TRAIL (SBE4-TRAIL) can reduce migration number (per mm^2^) and distance (mm) of tumor cells when compared with PBS (control), hAMSC-vector (vector) and hAMSC-SBE4-TRAIL+TβR inh (SBE4-TRAIL+TβR inh) group. **e** There was also a significant decrease in the tumor bulk volume by SBE4-TRAIL treatment as compared to other groups. **f** Representative pictures showed cleaved caspase-8 (purple) and td-tomato (red) staining of tumor satellites. Scale bar, 50 μm. **f**, **g** The SBE4-TRAIL treatment group exhibited a higher number of positive cleaved caspase-8 in the tumor satellites (beyond border of tumor mass) when compared with SBE4-TRAIL+TβR inh group. **h**, **i** The cleaved caspase-3 staining (purple) and td-tomato (red) assay also verified hAMSC-SBE4-TRAIL can induce apoptosis of human GBM satellites in GBM bearing mice. Scale bar, 50 μm. Error bars represent SEM. *p < 0.05, **p < 0.01, ***p < 0.001

Analysis of post-mortem tissue sections confirmed the reduction in migration and medium distance of tumor cell invasion as well as a maximum of tumor size when hAMSC-SBE4-TRAIL were injected. (Fig. 5a–e, no. of migration GBM cells, SBE4-TRAIL vs. control: 49.8 vs. 151.2, *p* < 0.001; migration distance of GBM cells, SBE4-TRAIL vs. control: 1.35 mm vs 3.40 mm, *p* < 0.001; maximum of tumor size, SBE4-TRAIL vs. control: 1.05 mm^2^ vs. 2.76 mm^2^, *p* < 0.01). Moreover, pharmacologic blockade of TβR signaling abolished the anti-cancer effects of the SBE4-TRAIL group as evidenced by reduced cleaved caspase-8 and cleaved caspase-3 compared to the group without the inhibitor treatment. Most importantly, dying GBM satellites of microscopic tumor satellites was significantly increased in SBE4-TRAIL group (Fig. 5f, g, cleaved caspase-8: *p* < 0.001; Fig. 5h, i, cleaved caspase-3: *p* < 0.001; Additional file 6: Figure S6A–C, TRAIL: *p* < 0.001). Their results reveal that hAMSC-SBE4-TRAIL was under the control of patient-derived GBM cells, as well as capable of inhibiting growth and inducing apoptosis in the tumor cells in vivo.

### hAMSC-SBE4-TRAIL by intracranial and intrathecal injection approach can prolong the survival of GBM bearing mice

**Fig. 6:**
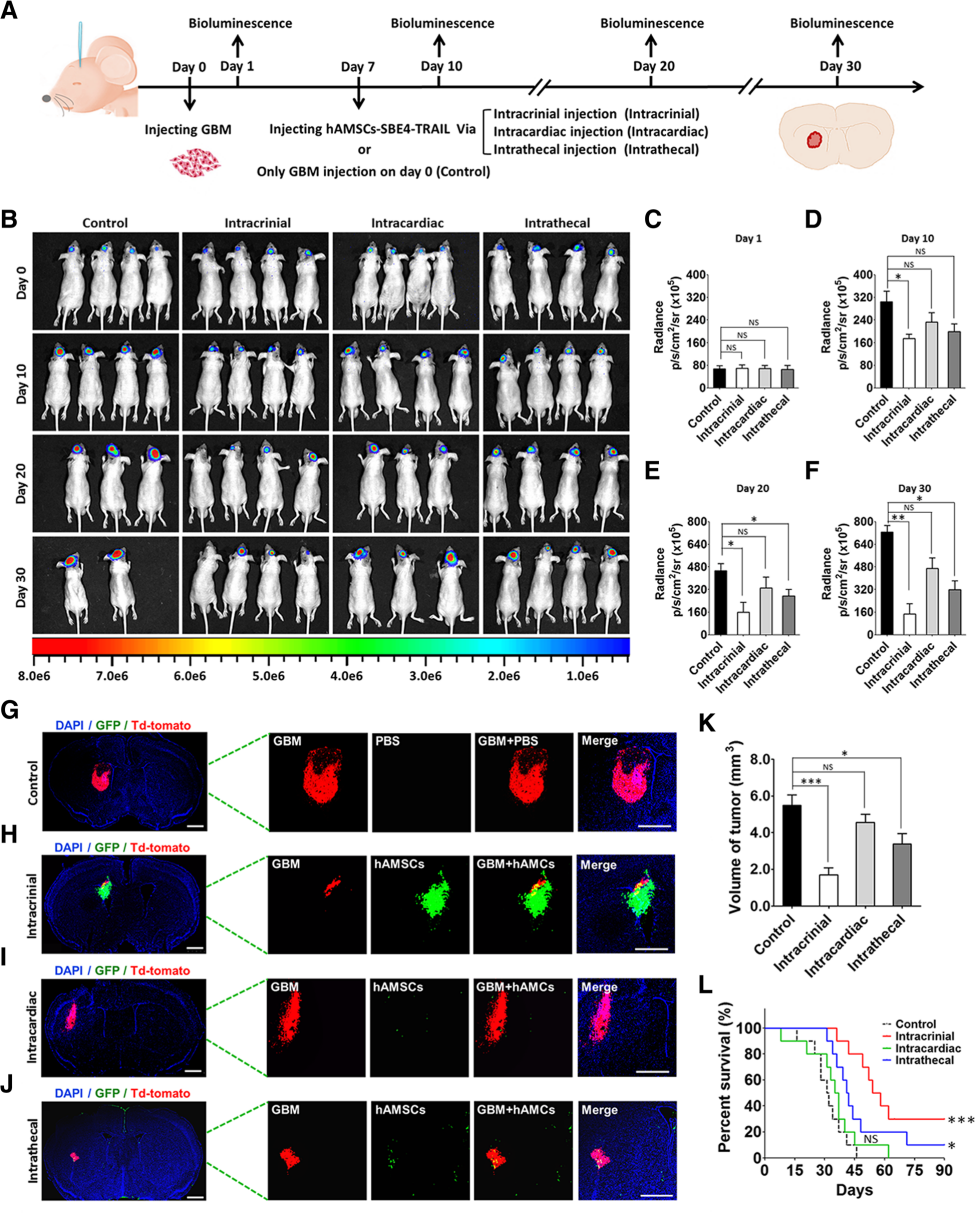
hAMSC-SBE4-TRAIL injection by intracranial and intrathecal approaches improves survival in GBM bearing mice. **a** Schematic of hAMSC-SEB4-TRAIL by different injection approach (hAMSC naive injection, control; intracranial injection, intracranial; intracardiac injection, intracardiac; and intrathecal injection, intrathecal) for GBM animal models. **b** Representative pictures showed bioluminescence for the GBM bearing mice on days 1, 10, 20, and 30 (*n* = 4 per group). **c–f** Bioluminescence signal radiance was significantly decreased in the intracranial group on days 10, 20, and 30 when compared with the control group. Meanwhile, there was also displayed inhibited effective on GBM growing in intrathecal group at days 20 and 30 as compared with control group. **g–j** Representative images showed the staining of GBM tumor mass (td-tomato, red) and the hAMSCs (GFP, green). Scale bar, 1 mm. **k** Analysis of post-mortem tissue sections confirmed the tumor volume decreased in the intracranial and intrathecal injection groups when compared with control group (*n* = 6 per group). **l** Kaplan-Meier survival analysis identified a significant median survival benefit in mice receiving hAMSC-SBE4-TRAIL by intracranial (*p* = 0.0003) and intrathecal (*p* = 0.0015) injection relative to the control group (*n* = 10 per group). Error bars represent SEM. *p < 0.05, **p < 0.01, ***p < 0.001. NS not significant

In order to investigate the efficiency of different therapeutic pathway, hAMSC-SBE4-TRAIL were delivery via three main approaches including in into the same location of tumor mass (intracranial injection), into the circulatory system (intracardiac injection), or into the cerebrospinal fluid system (intrathecal injection) in the GBM bearing mice (Fig. 6a). We found that the bioluminescent signal radiance was significantly lower in the intracranial and intrathecal groups while compared with the control group (Fig. 6b–f, intracranial vs control at days 10, 20, and 30, *p* < 0.05, *p* < 0.05, and *p* < 0.01; intrathecal vs control, days 20 and 30 at *p* < 0.05 and *p* < 0.05). Moreover, the tumor volumes of the intracranial and intrathecal injection group displayed significantly decreased values when compared with the control group (Fig. 6g–k, tumor volumes: control = 5.4 87 mm^3^, intracranial = 1.6 83 mm^3^, intracardiac = 4.558 mm^3^, and intrathecal = 3.667 mm^3^; intracranial vs control, *p* < 0.001, intrathecal vs control, *p* < 0.05, intracardiac vs. control, *p* = 0.08). Meanwhile, intracardiac groups did not exhibit a significant difference of tumor size and percent survival could be related to the effective concentration of these biologic vehicles in the central neural system. Most importantly, Kaplan-Meier survival analysis revealed a significant benefit of intracranial and intrathecal therapy as compared to the other groups (Fig. 6l, intracranial vs control, *p* < 0.001, intrathecal vs control, *p* < 0.05). These results support the conclusion that intracranial and intrathecal injection application routes are suitable for therapeutic stem cell placement to fight cancer.

## Discussion

The malignant nature of GBM has been attributed to its highly infiltrative capacity, rapid proliferation, self-renewal capacity, and its heterogeneous cell composition. GBM often utilizes white matter tracts as well as microvasculature basement membranes to migrate long distances, making complete surgical resection of tumor microscopic neoplastic satellites difficult, almost inevitably resulting in tumor recurrence in most of the cases [7, 14, 30]. Therefore, targeting and eliminating the GBM microsatellites and infiltrating tumor cells are of utmost significance in the treatment of this aggressive malignant primary intracranial neoplasm [6, 8, 31].

Stem cells have been described to be used as delivery vehicles for tumor therapy and—given their biologic, immune-compatible nature in autologous application—are considered to possess high potential for coherent clinical applicability. Recent studies emphasized that these biologic vehicles can be loaded with therapeutic genes to track microscopic tumor deposits and infiltrating tumor satellites in the brain that could not otherwise be removed by surgery [32, 33]. MSCs and NSCs have been proven to have strong tumor-tropic properties which have been exploited to fight neurological cancers [6, 11, 34]. Protnow et al. reported that 15 patients with recurrent GBMs received intracranial administration of NSCs to target brain tumors and locally produce chemotherapy at the time of resection or biopsy. The results showed that there was non-tumorigenic and no dose-limiting toxicity due to the stem cells [35, 36]. Recently, studies reported that MSCs were injected into the lateral ventricle for treatment of brain hemorrhage in preterm infants, and this phase I clinical trial showed MSCs are safe and feasible in the human brain [37]. More research is needed to assess the safety of this method to advance into clinical testing. Moreover, with the recent establishment of molecular subgroups of glioblastoma, it would be interesting to assess any subtype-specific vulnerabilities to hAMSC-mediated therapies [38, 39].

Engineering of MSCs to deliver therapies to cancer sites have emerged, including their use of secreting TRAIL to eradicate GBM as an attractive tool in neuro-oncology [12, 13, 24, 40]. Previous studies show that the treatment of TRAIL secreting by umbilical cord blood-derived mesenchymal stem cells (UCB-MSC) or bone marrow-derived mesenchymal stem cells (BM-MSC) has significant antitumor effects compared with adenoviral TRAIL gene therapy [16, 21, 41]. TRAIL is the key component of one of the most potent ligand-receptor-mediated cell death signaling pathways and therefore highly suitable to act as a suicide inducer as previously exploited [19, 42, 43]. Nevertheless, besides proving efficient tumor site homing, none of these studies addressed the potential risk of off-target therapy delivery due to the route of migration from the injection site toward the tumor nest. Provided that the disadvantages and potential complications of MSC that sustain TRAIL release into normal tissues have not been fully established, the clinical application of TRAIL delivery using stem cells is still limited [44–48].

**Fig. 7:**
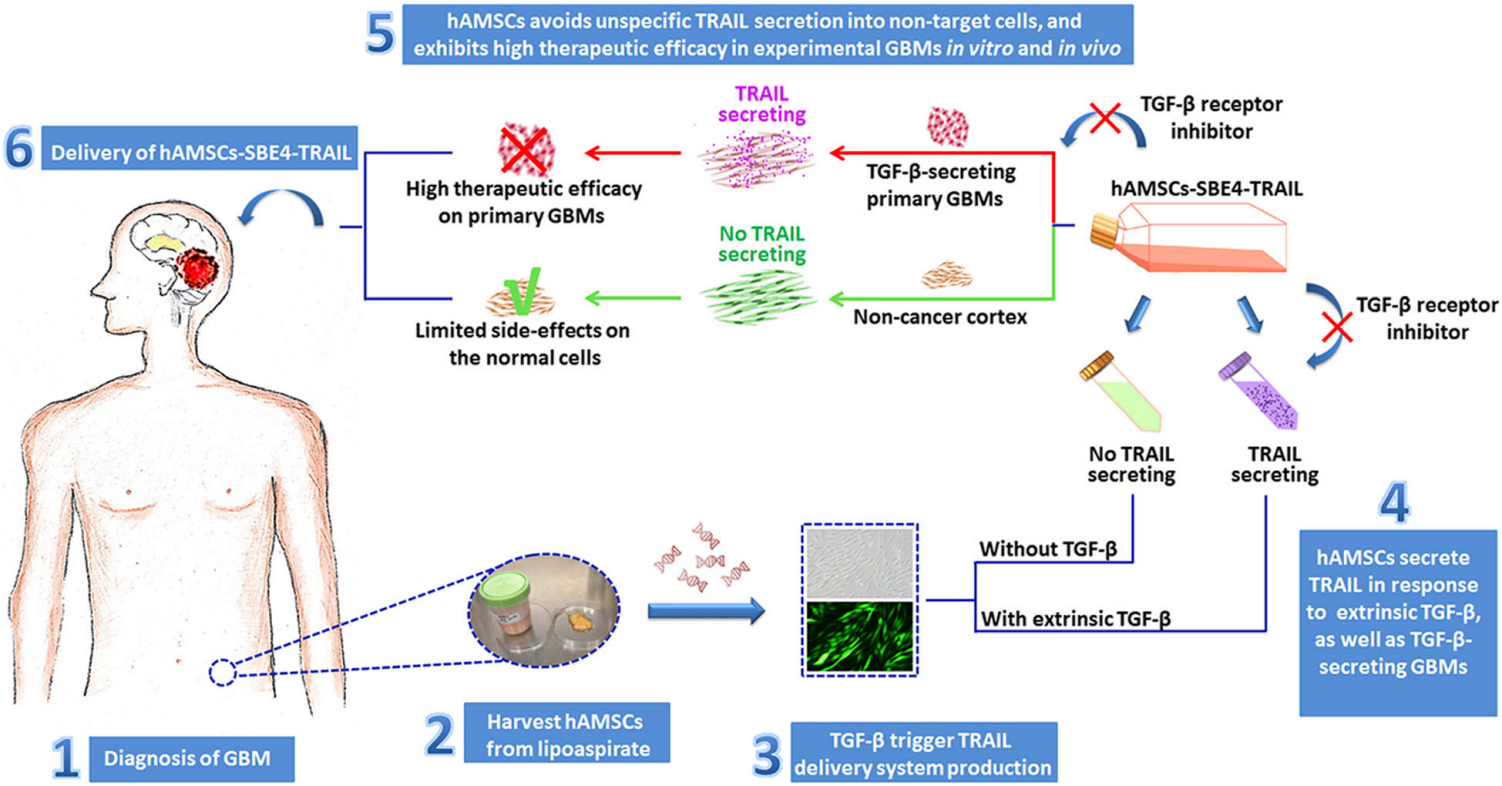
The hAMSC-SBE4-TRAIL delivery system has a potential clinical applications for GBM therapy. Our work sought to hAMSCs which augment the expression of TRAIL under the trigger of TGF-β-secreting primary GBMs and avoid unspecific TRAIL secretion into non-target cells. The results on the controlled release of the suicide gene delivery system demonstrate a significant improvement for the clinical utility of biological strategy to treat brain cancers, especially for the patients in whom tumor mass express elevated level of TGF-β

To address this, in our study, we developed a MSC-based treatment delivery system to fight an aggressive and disseminating tumor type in what its therapeutic activity is specifically induced once evaded in the periphery of the neoplasm. By genetically engineering an autologous cell source of *easy-to-harvest* and *easy-to-expand* fat MSCs [7, 16], that are highly sensitive to exogenous stimuli to induce the rapid secretion of a suicide gene payload (TRAIL), we are able to efficiently decrease GBM proliferation and migration while minimizing off-target effects on non-tumor cells. TGF-β was chosen as the signaling molecule of choice to induce the secretion of TRAIL given its high levels of expression in GBM cells as compared to any other brain cell type. Up to 70% of gliomas, especially GBM, express elevated levels of TGF-β which have been implicated in several tumor-related functions including proliferation, angiogenesis, stem-like cell properties, immunosuppression, invasiveness, and resistance to conventional therapies [27, 49–51]. Exploiting that TGF-β expression is highly upregulated in the GBMs compared with the normal cortex, it serves as a pathophysiological, molecular switch to modulate the cell-biologic behavior of the engineered delivery system. Our results show that hAMSC-SBE4-TRAIL released its therapeutic payload in response to TGF-β and they exhibit high safety criteria and selectively induce apoptosis in GBM cells while minimizing toxicity in the normal tissue (Fig. 7).

Although hAMSCs have been shown to migrate to a brain tumor in mouse models, the molecular mechanisms underlying the homing capacity of hAMSCs to lesions and tumors, particularly to GBM, have not been fully elucidated [14, 27, 52–54]. Our controlled conditions in the in vitro studies clearly support the notion that the effect is mediated by secreted factors of the GBM as conditioned media efficiently induced the tropism of hAMSCs. In wake of emerging concerns on the safety of using stem cells for therapy in medicine including oncology [11, 24, 45], our strategy utilizing intrinsic signals of the tumor microenvironment to induce the release of therapeutic load reveals significant improvement of target cell-specific reactivity as compared to previous studies using MSCs/NSCs as drug delivery systems. hAMSC-SBE4-TRAIL cause efficient tumor destruction in vitro and in vivo resulting in significant prolongation in overall survival in PDX model. In this study, we used TGF-β receptor inhibitor to neutralize TGF-β signaling to control hAMSCs secreting therapeutic gene. However, we did not show the effects of TGF-β receptor inhibitor on the target cells, or on the biological effects on the hAMSCs.

Utilizing surgical operation to remove the brain tumor mass and combining these biologic vehicles to eliminate micrometastatic nest may be given serially to maximize therapeutic benefit. Our results are in line with previous experimental and clinical observations that stem cells can be used to fight de novo brain tumor and disseminated tumor nests but extend their applicability/safety [13, 33, 36, 55]. Moreover, we provide preliminary evidence suggesting that the intrathecal injection could be an appropriate approach for stem cell therapy in the treatment of brain tumors. Meanwhile, this method could serve as a potential alternative approach when patients develop progressive weakness and intolerance to intracranial injection. Additionally, this study highlights hAMSC intrinsic migration capacity for malignant brain tumors and suggests that the TGF-β-induced TRAIL expression in hAMSCs may serve as a viable therapeutic option specifically among GBM patients with high levels of TGF-β expression.

## Conclusions

We propose fat-derived, inducible secretion of therapeutics using MSCs that may serve as a viable biologic treatment option to treat disseminating cancers with nominal off-target effects. hAMSC-SBE4-TRAIL are a potential treatment option to be tested in clinical trials to fight late-stage malignant brain cancers with disseminated microsatellite hot spots.

## Additional files

Additional file 1: **Figure S1.** Primary hAMSC characteristics. A, Representative images show the fluorescence photomicrographs of hAMSC-vector (vector) and hAMSC-SBE4-TRAIL (SBE4-TRAIL) scale bar, 100 μm. B, The passaging time of assayed primary cultured hAMSC-vector, hAMSC-SBE4-TRAIL and commercial hAMSC. C, Representative pictures show Vimentin staining of hAMSC-vector and hAMSC-SBE4-TRAIL. Scale bar, 50 μm. (TIF 2745 kb)

Additional file 2: **Figure S2.** The patient-derived GBM cells displayed a higher level of TGF-β_1_ and TGF-β_2_. A, Representative images show the white light and fluorescence photomicrographs of these patient-derived GBM cells (GBM025, GBM079, and GBM106). Scale bar, 50 μm. B and C, The concentration of TGF-β1 and TGF-β2 in patient-derived GBM cells as well as astrocytes were measured using an ELISA Kit. Error bars represent SEM. *p < 0.05, **p < 0.01, ***p < 0.001. (TIF 2651 kb)

Additional file 3: **Figure S3.** TRAIL expression of hAMSCs. A-D, The hAMSC-vector and hAMSC-SBE4-TRAIL were cultured in astrocyte-CM and GBM-CM (GBM025-CM, GBM079-CM, or GBM106-CM) for 24 h with or without TβR inh. Then, the TRAIL expression of these hAMSCs was confirmed by Western blot. (TIF 1648 kb)

Additional file 4: **Figure S4.** Proliferation assay of GBMs. A, Representative picture of Ki 67 (green) and DAPI (blue) staining of GBM025, GBM079, and GBM106. B-D, The GBMs (GBM025, GBM079, and GBM106) cultured in SBE4-TRAIL-C-CM had lower number of Ki 67 positive cells when compared with cultured in control-C-CM, vector-C-CM, and SBE4-TRAIL+TβR inh-C-CM conditions. Scale bar, 100 μm. Error bars represent SEM. *p < 0.05, **p < 0.01, ***p < 0.001. (TIF 3748 kb)

Additional file 5: **Figure S5.** GBMs co-cultured with hAMSCs. A, E and I, Representative images show the GBM cells (GBM025, GBM079, and GBM106; Td-tomato) co-cultured with hAMSCs (vector, SBE4-TRAIL and SBE4-TRAIL+TβR inh, GFP) for 0.5, 24, or 48 h. Scale bar, 100 μm. B-D, F-H, and J-L, While the GBM cells (GBM025, GBM079, and GBM106; Td-tomato) were co-cultured with hAMSC-SBE4-TRAIL (SBE4-TRAIL), the percentage of td-tomato positive cells displayed significant decrease. Error bars represent SEM. *p < 0.05, **p < 0.01, ***p < 0.001. (TIF 4857 kb)

Additional file 6: **Figure S6.** hAMSCs secreted TRAIL beyond the border of tumor mass. A and B, Representative images show GFP (green), Td-tomato (red), and TRAIL (purple) staining beyond the border of tumor mass. C, The TRAIL positive cells of hAMSCs were detected in the hAMSC-SBE4-TRAIL group, but were nearly absent in SBE4-TRAIL+TβR inh group. Scale bar, 50 μm. Error bars represent SEM. *p < 0.05, **p < 0.01, ***p < 0.001. (TIF 1795 kb)
