# Supplementary figures and images for: Exploiting tumor-intrinsic signals to induce mesenchymal stem cell-mediated suicide gene therapy to fight malignant glioma
Source: Stem Cell Res Ther. 2019 Mar 12;10:88. doi: 10.1186/s13287-019-1194-0 (PMC6417183; doi:10.1186/s13287-019-1194-0)

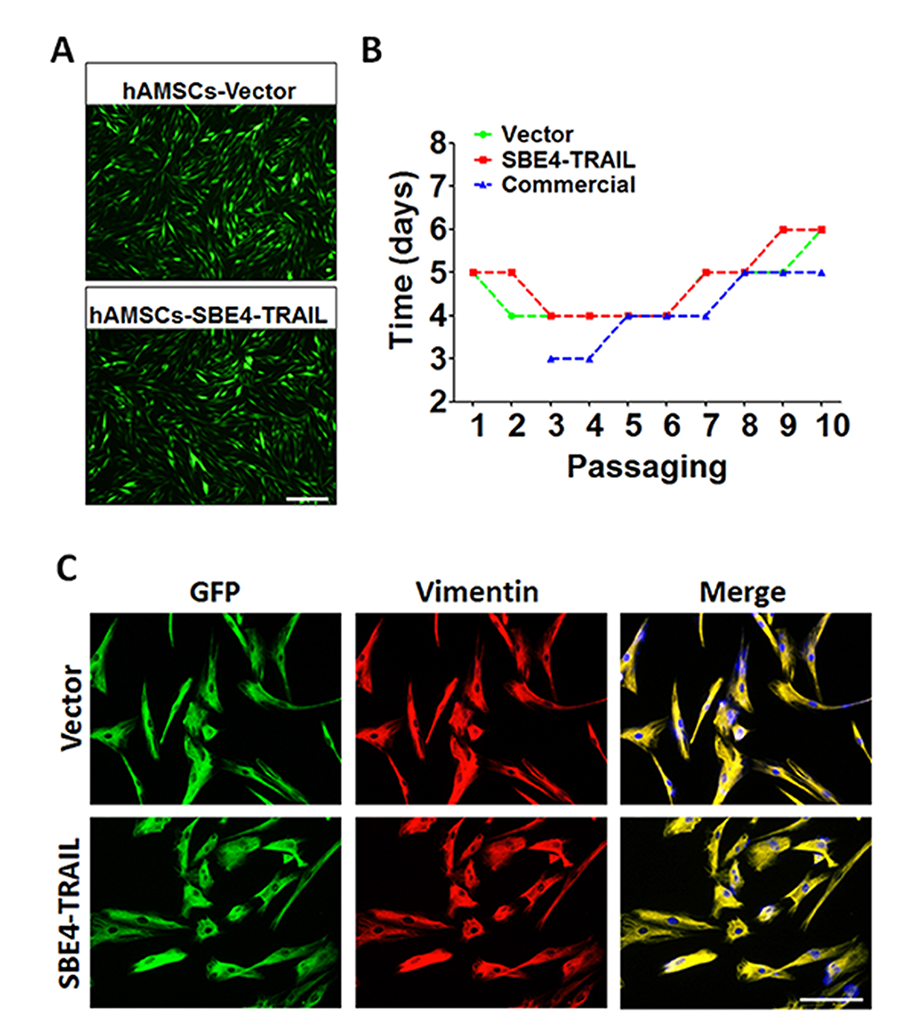

Supplement: Supplementary file 1 — Figure S1. Primary hAMSC characteristics. A, Representative images show the fluorescence photomicrographs of hAMSC-vector (vector) and hAMSC-SBE4-TRAIL (SBE4-TRAIL) scale bar, 100 μm. B, The passaging time of assayed primary cultured hAMSC-vector, hAMSC-SBE4-TRAIL and commercial hAMSC. C, Representative pictures show Vimentin staining of hAMSC-vector and hAMSC-SBE4-TRAIL. Scale bar, 50 μm. (TIF 2745 kb) [file 13287_2019_1194_MOESM1_ESM.tif]

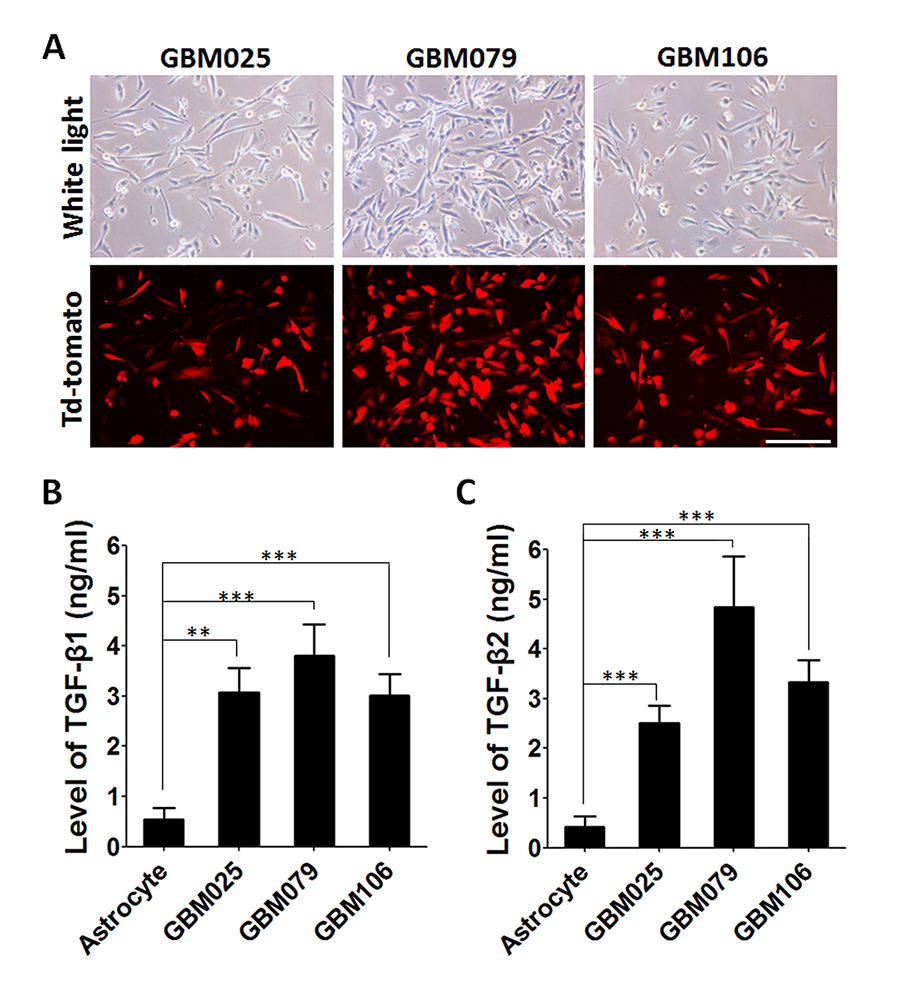

Supplement: Supplementary file 2 — Figure S2. The patient-derived GBM cells displayed a higher level of TGF-β1 and TGF-β2. A, Representative images show the white light and fluorescence photomicrographs of these patient-derived GBM cells (GBM025, GBM079, and GBM106). Scale bar, 50 μm. B and C, The concentration of TGF-β1 and TGF-β2 in patient-derived GBM cells as well as astrocytes were measured using an ELISA Kit. Error bars represent SEM. *p < 0.05, **p < 0.01, ***p < 0.001. (TIF 2651 kb) [file 13287_2019_1194_MOESM2_ESM.tif]

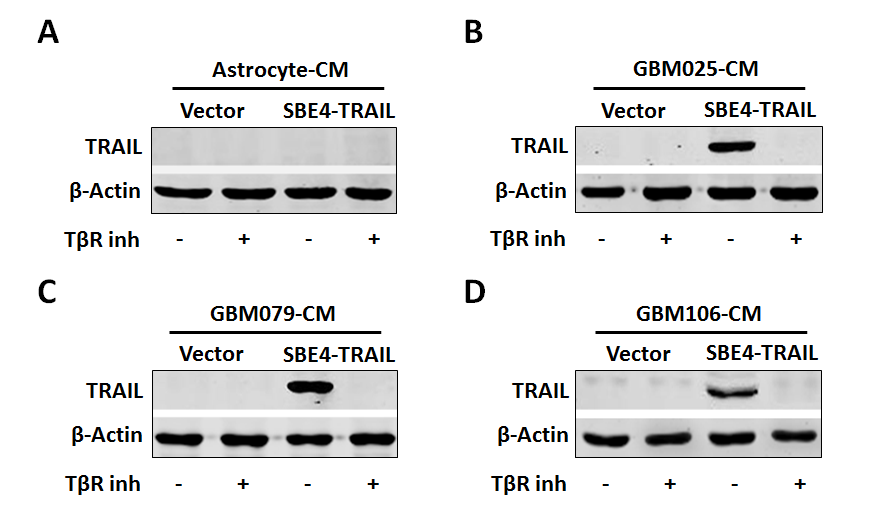

Supplement: Supplementary file 3 — Figure S3. TRAIL expression of hAMSCs. A-D, The hAMSC-vector and hAMSC-SBE4-TRAIL were cultured in astrocyte-CM and GBM-CM (GBM025-CM, GBM079-CM, or GBM106-CM) for 24 h with or without TβR inh. Then, the TRAIL expression of these hAMSCs was confirmed by Western blot. (TIF 1648 kb) [file 13287_2019_1194_MOESM3_ESM.tif]

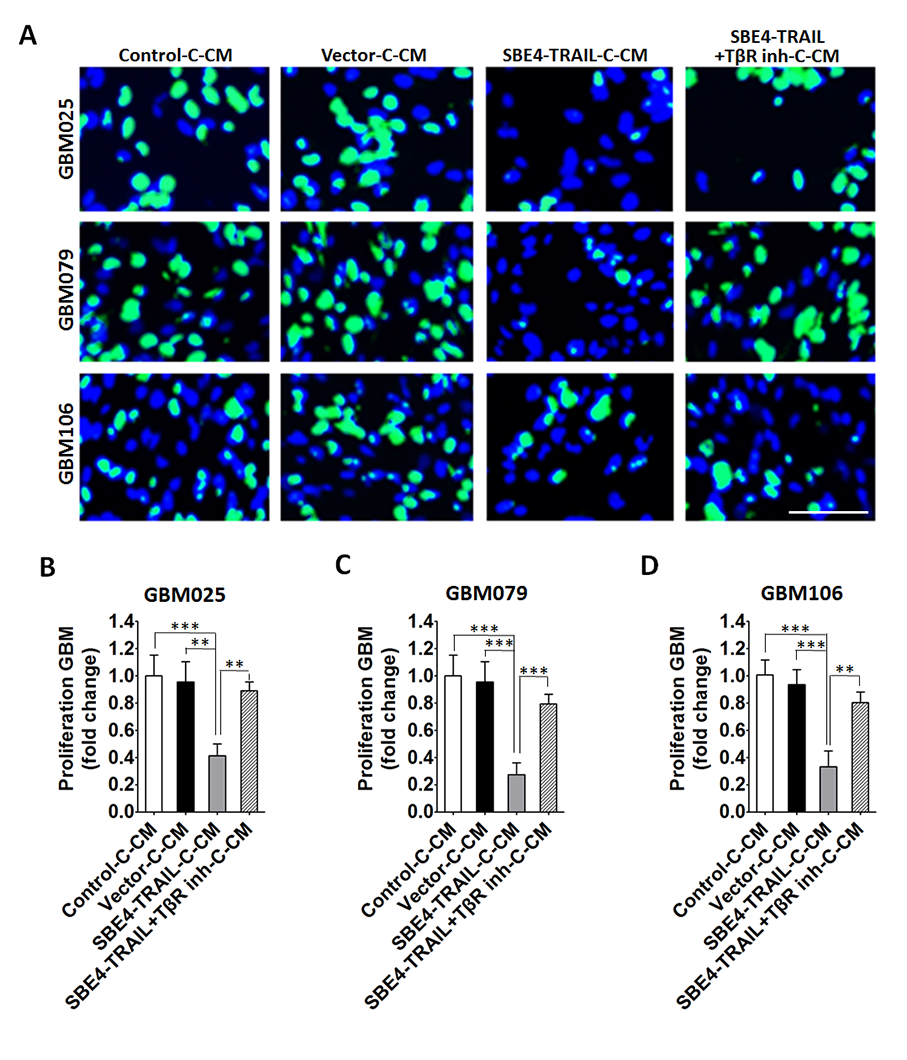

Supplement: Supplementary file 4 — Figure S4. Proliferation assay of GBMs. A, Representative picture of Ki 67 (green) and DAPI (blue) staining of GBM025, GBM079, and GBM106. B-D, The GBMs (GBM025, GBM079, and GBM106) cultured in SBE4-TRAIL-C-CM had lower number of Ki 67 positive cells when compared with cultured in control-C-CM, vector-C-CM, and SBE4-TRAIL+TβR inh-C-CM conditions. Scale bar, 100 μm. Error bars represent SEM. *p < 0.05, **p < 0.01, ***p < 0.001. (TIF 3748 kb) [file 13287_2019_1194_MOESM4_ESM.tif]

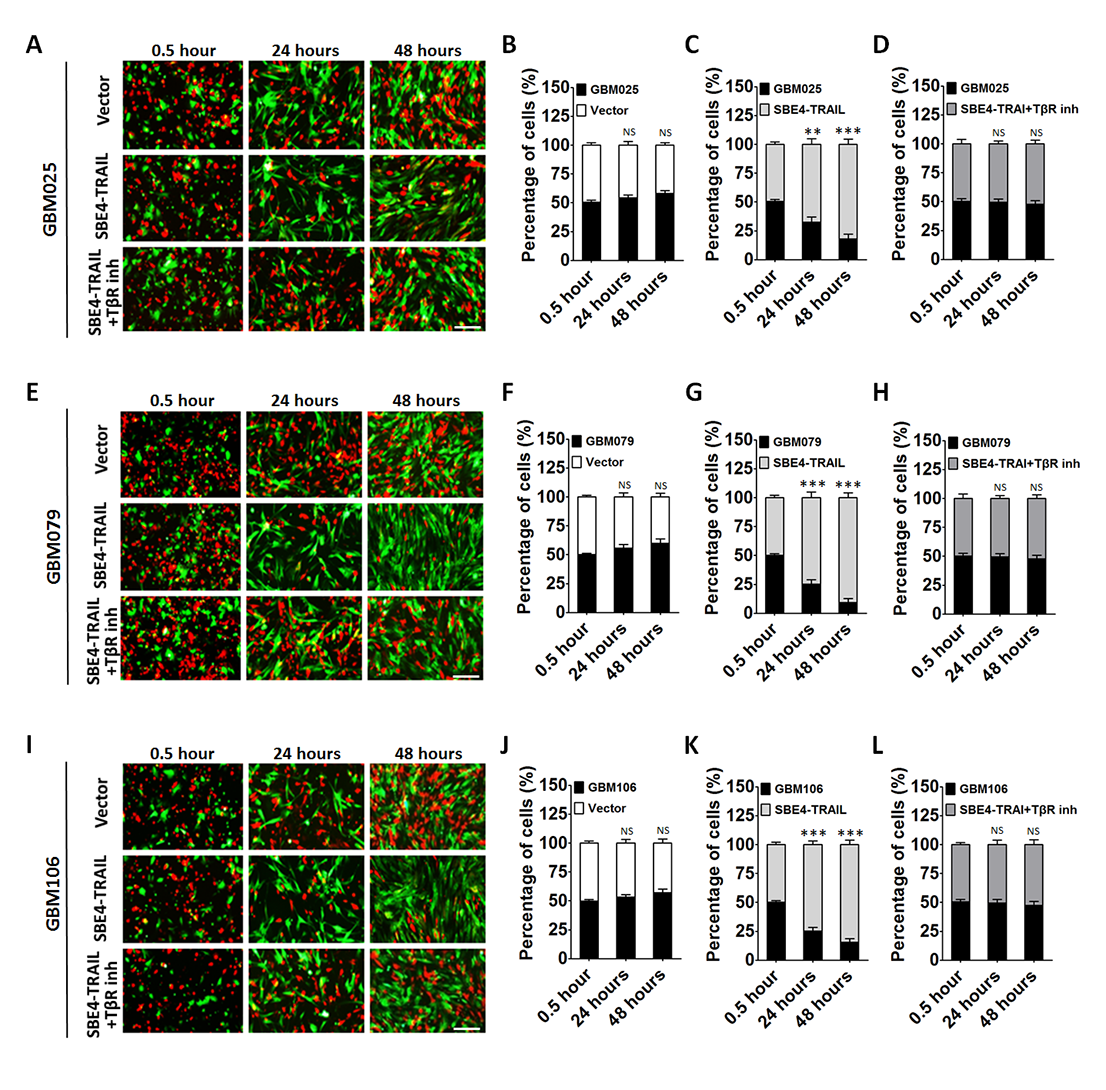

Supplement: Supplementary file 5 — Figure S5. GBMs co-cultured with hAMSCs. A, E and I, Representative images show the GBM cells (GBM025, GBM079, and GBM106; Td-tomato) co-cultured with hAMSCs (vector, SBE4-TRAIL and SBE4-TRAIL+TβR inh, GFP) for 0.5, 24, or 48 h. Scale bar, 100 μm. B-D, F-H, and J-L, While the GBM cells (GBM025, GBM079, and GBM106; Td-tomato) were co-cultured with hAMSC-SBE4-TRAIL (SBE4-TRAIL), the percentage of td-tomato positive cells displayed significant decrease. Error bars represent SEM. *p < 0.05, **p < 0.01, ***p < 0.001. (TIF 4857 kb) [file 13287_2019_1194_MOESM5_ESM.tif]

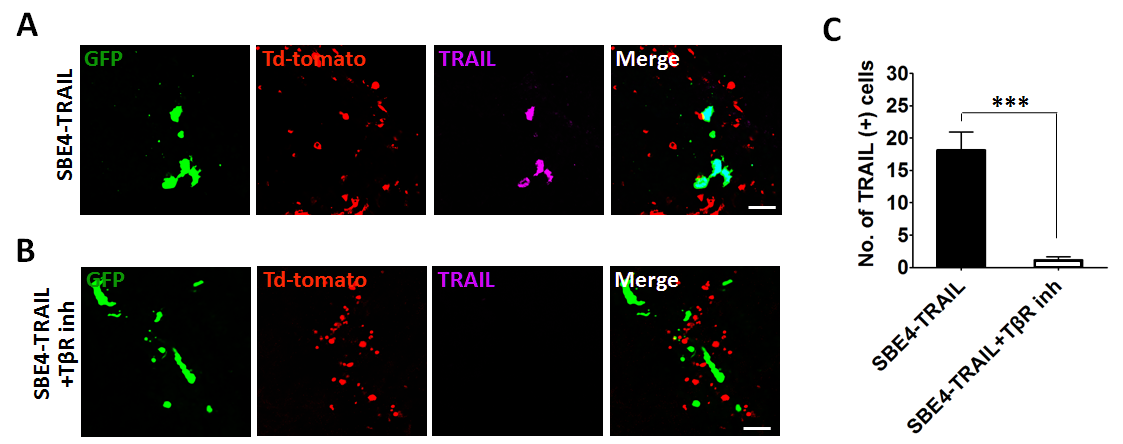

Supplement: Supplementary file 6 — Figure S6. hAMSCs secreted TRAIL beyond the border of tumor mass. A and B, Representative images show GFP (green), Td-tomato (red), and TRAIL (purple) staining beyond the border of tumor mass. C, The TRAIL positive cells of hAMSCs were detected in the hAMSC-SBE4-TRAIL group, but were nearly absent in SBE4-TRAIL+TβR inh group. Scale bar, 50 μm. Error bars represent SEM. *p < 0.05, **p < 0.01, ***p < 0.001. (TIF 1795 kb) [file 13287_2019_1194_MOESM6_ESM.tif]
